# Development and Validation of A 48-Target Analytical Method for High-throughput Monitoring of Genetically Modified Organisms

**DOI:** 10.1038/srep07616

**Published:** 2015-01-05

**Authors:** Xiaofei Li, Yuhua Wu, Jun Li, Yunjing Li, Likun Long, Feiwu Li, Gang Wu

**Affiliations:** 1 Key Laboratory of Oil Crop Biology, Ministry of Agriculture, Oil Crops Research Institute, Chinese Academy of Agricultural Sciences, No. 2 Xudong 2nd Road, Wuhan 430062, People's Republic of China; 2 Supervision and Test Center (Wuhan) for Environmental Safety of Genetically Modified Plants, Ministry of Agriculture, No. 2 Xudong 2nd Road, Wuhan 430062, People's Republic of China; 3 Agro-Biotechnology Research Institute, Jilin Academy of Agricultural Sciences, No.1363 Caiyu Avenue, Changchun 130033, People's Republic of China

## Abstract

The rapid increase in the number of genetically modified (GM) varieties has led to a demand for high-throughput methods to detect genetically modified organisms (GMOs). We describe a new dynamic array-based high throughput method to simultaneously detect 48 targets in 48 samples on a Fludigm system. The test targets included species-specific genes, common screening elements, most of the Chinese-approved GM events, and several unapproved events. The 48 TaqMan assays successfully amplified products from both single-event samples and complex samples with a GMO DNA amount of 0.05 ng, and displayed high specificity. To improve the sensitivity of detection, a preamplification step for 48 pooled targets was added to enrich the amount of template before performing dynamic chip assays. This dynamic chip-based method allowed the synchronous high-throughput detection of multiple targets in multiple samples. Thus, it represents an efficient, qualitative method for GMO multi-detection.

With the rapid development of biotechnology, the production of genetically modified (GM) crops has increased continuously since the commercial planting of the first approved GM variety in 1996. According to the International Service for the Acquisition of Agri-Biotech Applications (ISAAA), 27 types of plants and 336 transgenic varieties had been developed and commercialized worldwide by the end of 2013, and the planting area of GM crops was greater than 175 million hectares in 27 countries^1^. Many new GM varieties are entering field trials or nearing environmental release. In many countries, the government and the public have a degree of distrust for genetically modified organisms (GMOs) because of perceived risks to food safety and the environment^2^. To protect the consumer's right to information, many countries have developed legislation to regulate and track the presence of GMOs in feed and foodstuffs^3^,^4^.

To comply with GMO legislation and to ensure product legality and traceability, effective and accurate analytical methods are essential for GMO screening, identification, and quantification. The DNA-based polymerase chain reaction (PCR) technique is the most widely accepted GMO detection method, and has been classified into screening, gene-specific, construct-specific, and transgenic event-specific methods according to the level of specificity^3^,^5^. During GMO detection, a series of PCRs are performed sequentially to determine the identity of GMO ingredients (authorized or unauthorized) in the test samples, first screening for elements generally used in GMOs, and then for event-specific sequences. Because of the large number and complexity of GM varieties, it is generally not feasible to conduct traditional single-target PCRs for all possible elements and events. Also, the traditional detection strategy is time-consuming, laborious, and costly^6^. Because of the rapid increase in the number of new GMOs, there is a need for high-throughput screening and identification methods to simultaneously detect multiple targets, in multiple samples, in a single experiment. Many multiple-target detection methods for GMO screening and identification have been reported, including multiplex PCR, multiplex PCR-based techniques, and the ready-to-use real time PCR matrix^3^,^7^,^8^,^9^,^10^.

The objective of multiplex PCR is to detect multiple targets simultaneously in a single reaction. Multiplex PCR often requires careful optimization of the composition and concentration of reagents and primers^7^,^11^. Conventional multiplex PCR can detect only five to six targets because of the poor separation of the PCR products in ordinary electrophoresis^8^. The multiplexing capability of multiplex real-time PCR is restricted by the number of channels in the real-time PCR thermal cycler, and by the number of available fluorescent dyes^12^,^13^,^14^. To improve the multiplexing capability in GMO detection, new detection strategies have been introduced and applied to GMO diagnostics. Such strategies combine multiplex PCR with capillary gel electrophoresis (CGE) or microarray hybridization. In particular, DNA microarray assays have shown considerable potential for the simultaneous analysis of thousands of different targets^10^,^15^,^16^,^17^,^18^,^19^. However, in a practical context, the target throughput for both the multiplex PCR-based CGE method and the microarray method is often limited by the multiplicities of multiplex PCR^15^,^20^,^21^. Other limitations of multiplex PCR include the preferential amplification of partial targets, and non-specific background amplification resulting from interference of multiple primer combinations in a single reaction^22^,^23^. To overcome the defects of multiplex PCR associated with end-point PCR technology, an initial multiplex preamplification step with a low concentration of initial primers and a low PCR amplification cycle number have been used to increase the amount of template. This strategy has been used in several high-throughput DNA detection methods, including nucleic acid sequence-based amplification (NASBA) implemented microarray analysis (NAIMA), multiplex quantitative DNA array-based PCR (MQDA-PCR), and multiplex microdroplet PCR implemented capillary gel electrophoresis (MPIC)^9^,^20^,^24^. A recently reported high-throughput method, MACRO, consists of multiplex amplification on a chip, and uses an oligo microarray for readouts of multiple amplicons. This method simultaneously detected 91 targets^25^. In general, microarray-based analytical approaches are very complex and expensive, and are therefore impractical for GMO detection.

The ready-to-use real-time PCR matrix is a 96-well or 384-well prespotted plate containing lyophilized primers and probes, allowing the simultaneous detection of multiple targets^26^,^27^. The real-time PCR-based ready-to-use 96-well plate was developed by the Joint Research Centre to analyze two samples per plate, detecting 48 individual targets^26^. Then, a multiplex real-time PCR method was established to simultaneously perform 24 multiplex real-time PCRs on a 384-well ready-to-use plate, allowing the detection of 47 targets in seven samples in duplicate^27^. Compared with multiplex PCR, the use of prespotted plates is more flexible, and testing parameters can be added or removed as needed without the need to re-optimize the entire system. However, the ready-to-use multi-target analytical system can analyze only a few samples in a single experiment because of the trade-off between the target throughput and sample throughput.

The Fluidigm system with an integrated fluidic circuit (IFC) chip is a new high-throughput detection platform. The IFC chip used in the Fluidigm system includes digital and dynamic arrays. The digital arrays allow the precise quantification of nucleic acids. This system has been used to measure variations in copy number and to perform molecular diagnostics of lung cancer, gastrointestinal cancer, and other diseases^28^,^29^,^30^,^31^. In GMO analysis, digital array PCR has been used to assess detection limits^32^. Microfluidic dynamic array technology has been used in a number of high-throughput analyses such as gene expression analysis, microRNA expression analysis, and single cell gene expression analysis^33^,^34^,^35^,^36^. Currently, the IFC chips available for dynamic analysis include the 48.48 (48 samples*48 assays), the 96.96 (96 samples*96 assays), and the 192.24 (192 samples*24 assays). Compared with other methods, the dynamic chip method can maintain compatibility by using validated PCR protocols in a high-throughput format. In 2014, Brod et al. first reported a high throughput method targeting 28 GMO elements using dynamic chips^37^.

Since 1994, 62 countries and regions (35 countries + 27 member states of the EU) have granted regulatory approval for 336 transgenic events for food and/or feed use and for environmental release or planting. The type and number of approved events differs among different countries^1^. At present, 37 kinds of GM crops have been approved by China for import as raw materials for processing. During GMO detection, the more parameters are tested, the higher the chance of detecting a GMO ingredient. Therefore, we developed a 48-plex higher-throughput GMO detection method based on validated real-time PCR methods and the dynamic IFC chip. This method allowed the simultaneous high-throughput detection of multiple targets in multiple samples. The targets included species-specific genes, regulatory elements, target genes, most of the Chinese approved transgenic events, and several unapproved events.

## Results

### Selection of targets

We selected detection targets based on the current Chinese GMO regulations. The detection targets included seven screening elements (P-CaMV35S, P-FMV35S, T-NOS, *NPTII*, *Cry1Ab*, *Bar*, and *Pat*), 36 transgenic events (transgenic cotton MON531, MON88913, MON1445, MON15985, LLCotton25, GHB614; transgenic maize 3272, 59122, Bt176, Bt11, GA21, MIR162, MIR604, MON810, MON863, MON88017, MON89034, NK603, T25, TC1507; transgenic rapeseed MS1, Topas 19/2, OXY-235, MS8, RF3, RT73, T45; transgenic soybean 40-3-2, A2704-12, MON89788, MON87701, DP-356043, A5547-127, CV-127, DP-305423-1; transgenic rice TT51-1) and 5 reference genes (cotton, maize, rapeseed, rice, and soybean). The selected targets covered either 100% (37/37) approved transgenic varieties or three unapproved events TT51-1, MIR162, and A5547-127 in China. Out of the 37 Chinese approved GM varieties, 31 could be detected based on the above seven screening elements; the six events that could not be detected based on these elements were GM soybean MON89788, MON87701, DP356043, DP305423, BPS-CV127-9 and GM cotton GHB614. These lines do not harbour commonly used screening targets, and so they must be analyzed using event-specific methods.

Data on the presence of regulatory elements and genes in GM crops in the GM crops database indicate that these 43 foreign DNA targets could be used to detect most of the released transgenic events, including 100% (16/16) of transgenic rapeseed, 96.4% (27/28) of transgenic maize, 92.3% (13/14) of transgenic cotton, 100% (2/2) of transgenic rice, 84.6% (11/13) of transgenic soybean, and 93% (28/30) of other transgenic crops. Therefore, the targets selected in this study not only allow detection and identification of Chinese authorized GM crops, but also allow the detection of unauthorized transgenic events in test samples.

### Validation of real-time PCR methods

We selected 36 event-specific methods to establish the high-throughput system. The MON87701 soybean was not available, and so the specificity and sensitivity of the MON87701-specfic method could not be validated. To test the specificity of the other 35 event-specific methods, two samples were prepared for each TaqMan assay. One was the DNA solution from the corresponding transgenic event; and the other was a mixture of 34 events, that is, all events except for the corresponding event. Real-time PCR reactions were run with these samples as the template for each TaqMan assay, in triplicate. The amplification results showed that these 35 event-specific methods identified specific transgenic events, and no typical amplification plots were produced from mixed samples. The results of the specificity test also indicated that there was no cross-contamination among different events during genomic DNA extraction. The specificity of seven screening methods was also validated by amplifying the corresponding targets in 35 transgenic events, displaying expected specificity.

To test the sensitivity of the detection methods, the genomic DNA was diluted to 10 and 5 copies/µL. Ten copies of target DNA could be consistently detected for all of the real-time PCR methods. These results suggested that the primer/probe sets used in this study had high specificity and good sensitivity, and were suitable for use in subsequent experiments.

### Preamplification

**Figure 1:**
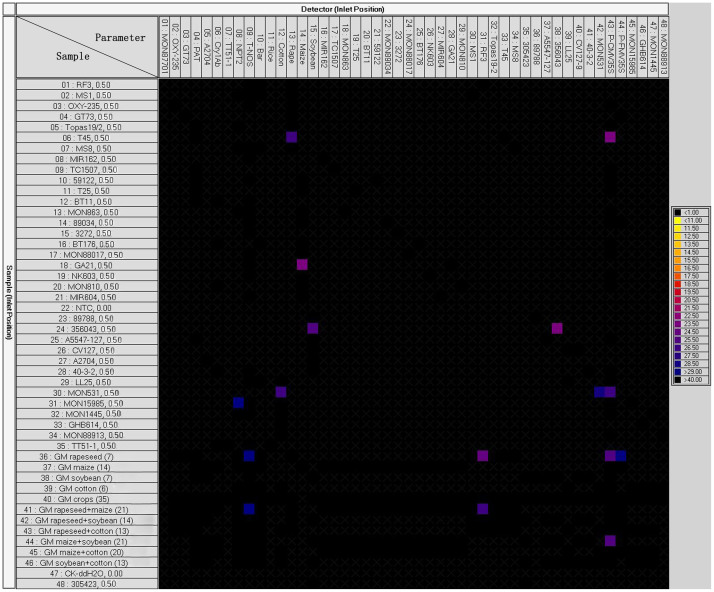
Panel readouts from the dynamic chip using 0.5 ng GM DNA solutions from test samples as templates. Coloured squares correspond to positive hits on the chip, black squares indicate chambers with no amplification.

Only a few reaction chambers produced a fluorescence signal when the 35 single-event DNA solutions at 0.5 ng/μL level and 11 complex samples (S36–S46) were directly used as DNA templates to run dynamic chip assays on the Biomark system (Fig. 1). Based on the genome size of the five crop species^38^, the template concentration, and the chamber volume (9 nL) of the 48.48 dynamic chips, the copy number of haploid genomes per reaction chamber was determined to be 2.2 for rice, 0.3 for maize, 0.4 for cotton, and 1.0 for rapeseed and soybean. These results indicated that the amount of template was too low to generate an amplification plot. Therefore, the sensitivity of more than 0.5 ng GM DNA for the dynamic chip was insufficient for real GMO samples.

To increase the sensitivity of the dynamic chip system, preamplification reactions were performed with a PreAmp Master Mix kit to increase the amount of template. An average of three PCR templates per well is the minimum amount to detect the presence of the target at the 95% confidence level, and for the samples of 0.1% level the copy number of the reference gene target needs to be amplified to at least 3000. Therefore, the preamplification would require at least 8–9 cycles to meet the requirements for sensitivity. Considering PCR efficiency is usually less than 100% in practice, a 14-cycle preamplification was performed, as recommended by the manufacturer.

To check the preamplification results, using 0.5 ng of GM DNA and preamplification products from eight transgenic events in five crops as templates, partial TaqMan assays were carried out to compare the Ct values of templates before and after preamplification (Table 1). The Ct values of detection targets after preamplification were significantly decreased by an average of 10.1, compared with those before preamplification (Table 1). This result indicated that the abundance of targets in the template was greatly increased. However, the delta Ct values between the original template and preamplified template differed among the targets (Table 1). This result suggested that the ratio of different targets after preamplification deviated from that before preamplification, and the preamplification products were not suitable for quantitative analysis of GMO content in original samples. Consequently, we used preamplified products only for qualitative detection in subsequent analyses.

### Sensitivity of dynamic chip assays

**Figure 2:**
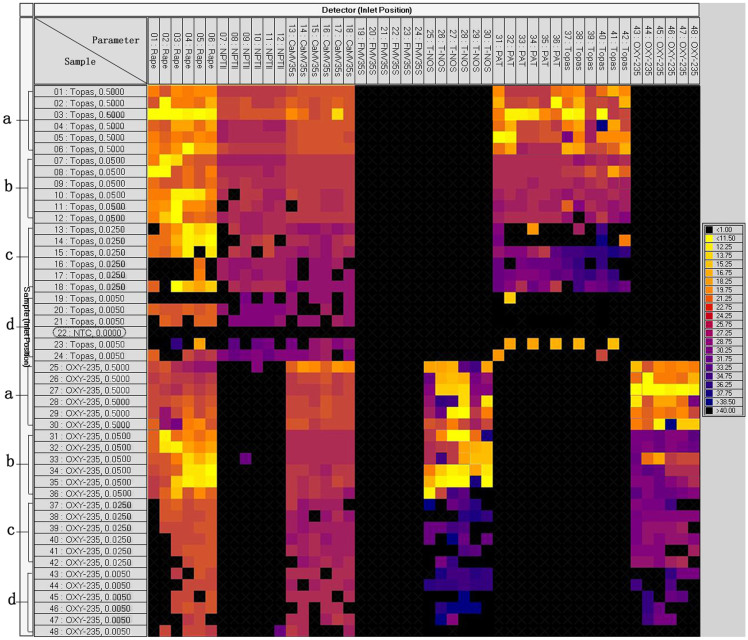
Analytical sensitivity of dynamic chip assays in detecting serially diluted genomic DNA solutions from Topas 19/2 and OXY-235 events. a–d correspond to GMO DNA amount of 0.5 ng, 0.05 ng, 0.025 ng, and 0.005 ng, respectively. Coloured chambers indicate positive signals; black chambers indicate negative signals.

The genomic DNA from transgenic rapeseed Topas 19/2 and OXY-235 were serially diluted to 0.5, 0.05, 0.025 and 0.005 ng/μL using salmon sperm DNA. The diluted DNA samples were used as templates in the preamplification reactions with pooled TaqMan assay mixture. After preamplification, the diluted preamplification products were used in real-time PCR analyses on dynamic chips to test the sensitivity of the dynamic chip assays. In this run, there were six replicates of seven samples, five replicates of 0.005 ng Topas 19/2, and one NTC control (No 22 sample). Eight targets including the rapeseed reference gene, *NPTII*, CaMV35S, FMV35S, T-NOS, *PAT*, Topas 19/2, and OXY-235, were assayed with six parallels per target (Fig. 2). As shown in Fig. 2, the NTC produced no amplification signal, and 0.5 ng DNA of both Topas 19/2 and OXY-235 generated the expected results with true positive and negative signals, respectively. The 0.05 ng DNA of both Topas 19/2 and OXY-235 showed near-perfect results with four false negatives for Topas 19/2 and one false positive for OXY-235. For the 0.025 ng and 0.005 ng samples, the number of chambers with a false negative signal increased as the GMO content decreased. The Fluidigm run results suggested that the 0.05 ng of GMO DNA can be consistently detected on a dynamic chip after preamplification, corresponding to 44 copies of the targets. The preamplification proved to be a practical method to increase the size of the template when analyzing real samples. The variations in Ct values were relatively large among parallels. This result suggested that the distribution of template was not uniform among chambers, resulting in different signal intensities.

### Validation of high-throughput PCR on dynamic chips

**Figure 3:**
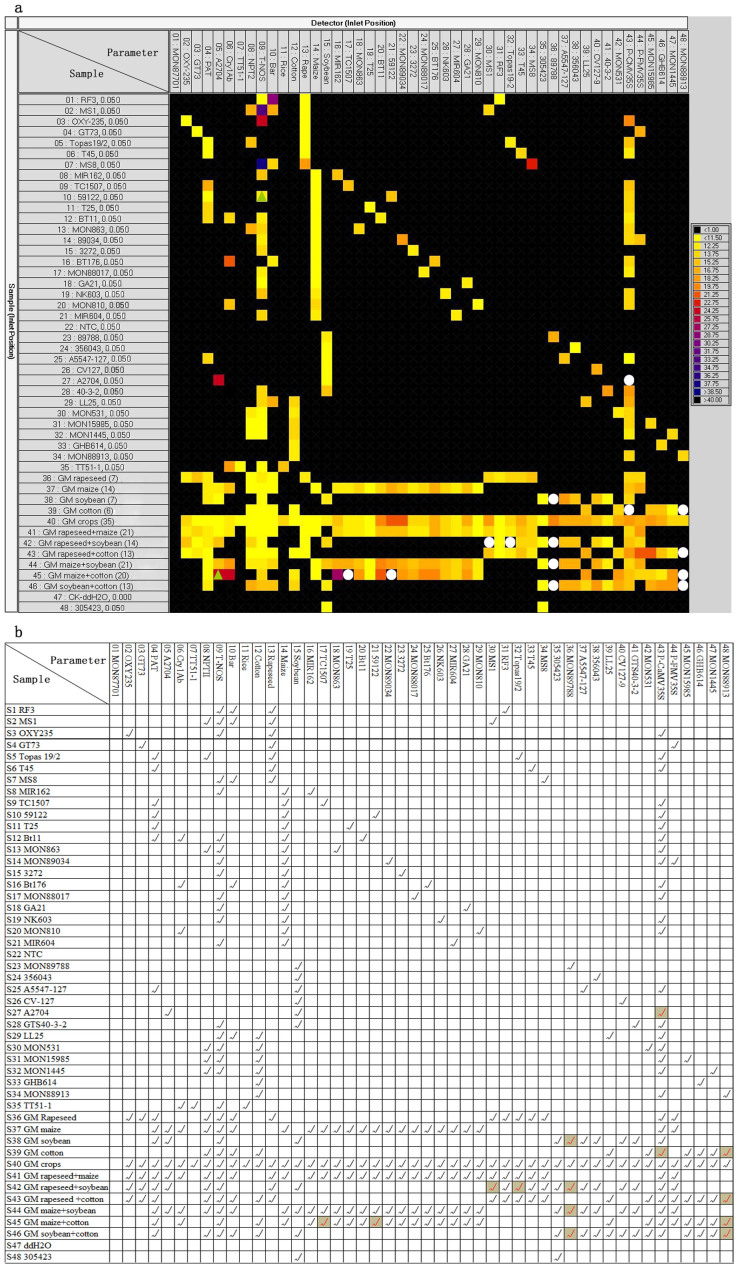
“Heat map” of 48.48 dynamic array and tabulated data of presence/absence of test targets in samples. (a), Heat map showing TaqMan PCR amplification in a dynamic array chip panel, calculated using Q-PCR Analysis software. Coloured squares indicate positive chambers. Colours on map correspond to Ct values (see colour-coded legend on the right). Green triangles indicate false positive chambers; white circles indicate false negative chambers. (b), Presence of detection targets in the test samples. Tick symbol (✓) indicates presence of detection targets, grey colour indicates undetected targets in dynamic chip assays.

The Fluidigm 48.48 real-time PCR run was performed to simultaneously detect 48 targets in 48 preamplified samples. Table 2 shows information on the 48 targets. The 48 samples included an NTC control and a blank control, which allowed us to assess the reliability of the dynamic chip runs; 35 single-event samples corresponding to 35 transgenic events at 0.05 ng/µL concentration, and 11 DNA mixtures simulating gene-stacking and mixed samples in practice. The NTC control and blank control (S22 and S47) did not generate any signals, and the MON87701 assay did not generate a signal because of the absence of a MON87701 event. However, most of the TaqMan assays successfully amplified targets from samples known to contain these targets (Fig. 3A). The tabulated data on the presence/absence of targets in samples (Fig. 3B) shows that 33 single-event samples gave excellent amplification results. Sample S10 (59122) gave a false positive result in the chamber corresponding to the T-NOS assay, and S27 (A2704) gave a false negative result in the chamber corresponding to the P-CaMV35S assay. The five samples that contained no common screening targets [S23 (MON89788), S24 (DP356043), S26 (CV-127), S33 (GHB614), and S48 (DP305423)] were accurately identified by event-specific assays. The sensitivity of dynamic chip assays were further demonstrated to be at least 0.05 ng DNA amount for each GM event, corresponding to 19 copies of haploid genome for maize, 25 copies for cotton, 112 copies for rice, 44 copies for rapeseed and soybean. For the 11 complex multiple-event samples, four samples (S36, S37, S40 and S41) produced perfect results, and the other seven samples (S38, S39, S42–S46) produced near-perfect results with minor incidences of false negatives (Fig. 3). This dynamic chip assay exactly detected 35 different events (regulatory elements, functional genes, and GM events) in the mixed sample S40. The successful detection of GM events in complex samples further indicated that the preamplification procedure efficiently increased the amounts of multiple targets.

## Discussion

The dynamic chip-based method developed in this study allowed the simultaneous detection of 48 targets in 48 samples on a Biomark system. In this assay false positive results were rarely observed, indicating that there was no cross-contamination during the preparation of test samples and TaqMan assay mixtures. In practice, sample repeats should be set to rule out the false positive or negative results. If inconsistent results were observed between the repeats, the traditional real-time PCR assays should be performed as a followup to further confirm the test results. The validation results demonstrated that this method could consistently detect samples with a GM DNA amount as low as 0.05 ng, and was capable of detecting a variety of GM ingredients in a complex mixture. The strategy of simultaneous detection of multiple targets integrates the GMO screening and identification steps. Therefore, this method can detect not only authorized GMOs, but also new GM events or unauthorized GMOs. The next goal of this research is to establish a 96.96 dynamic chip-based higher-throughput detection method to simultaneously detect and identify 96 targets in 96 samples. In this analysis, the targets would cover major screening elements, all approved GM events, and published unapproved GM events.

Compared with previously established high-throughput methods, our new method has the advantages of flexible throughput, high efficiency, relatively low cost, intuitive results, and suitability for detecting targets in complex samples. The high-throughput method could be used to efficiently and accurately identify GMOs in samples with greatly increased target and sample throughputs. Therefore, this method will be useful for detecting multiple GM events, allowing compliance with GMO regulations in China and other countries.

## Methods

### Plant materials

Transgenic materials and non-transgenic seeds were identified and collected by our own GMO detection laboratories. The materials included 14 transgenic maize lines (3272, 59122, Bt176, Bt11, GA21, MIR162, MIR604, MON810, MON863, MON88017, MON89034, NK603, T25, and TC1507), 7 transgenic soybean lines (40-3-2, A2704-12, MON89788, DP-356043, A5547-127, CV-127, and DP-305423-1), 7 transgenic rapeseed lines (MS1, Topas 19/2, OXY-235, MS8, RF3, RT73, and T45), 1 transgenic rice line (TT51-1) and 6 transgenic cotton lines (MON531, MON88913, MON1445, MON15985, LLCotton25, and GHB614).

### DNA extraction

A DNeasy Plant Mini Kit (QIAGEN, Hilden, Germany) was used to extract and purify genomic DNA from seeds or seed powders. DNA purity was checked with a NanoDrop 2000 spectrophotometer (Thermo, Wilmington, DE, USA). The DNA concentrations in samples were determined using a Qubit® 2.0 Fluorometer (Life Technologies, Carlsbad, CA USA) with a Qubit® dsDNA BR Assay Kit (Life Technologies). The purified genomic DNA were adjusted to the same concentration of 50 ng/μL using 1 × TE buffer (1 mmol/L Tris, 0.01 mmol/L EDTA, pH 8.0), and were stored at −20°C until use.

### Preparation of test samples

The genomic DNA solutions extracted from 35 transgenic lines were diluted to 5.0 ng/μL, 0.5 ng/μL, and 0.05 ng/μL levels using salmon sperm DNA, the total DNA concentration for all samples were kept at 50 ng/μL. The DNA solutions at a concentration of 0.05 ng/μL were directly used as test samples and labelled as S1–S21, S23–S35, and S48. The DNA solutions at 0.5 ng/μL concentration were used to prepare mixtures of rapeseed, maize, soybean, and cotton DNA by mixing equal volumes of genomic DNA from each line, and labelled as S36–S39. Equal volumes of the DNA solutions at a concentration of 5 ng/μL were mixed together to prepare a complex sample, S40, containing 35 events from five crops, and mixed to prepare six other complex samples containing multiple events (S41-S46) from two GM crops: S41 contained 7 rapeseed events and 14 maize events, S42 contained 7 rapeseed events and 7 soybean events, S43 contained 7 rapeseed events and 6 cotton events, S44 contained 14 maize events and 7 soybean events, S45 contained 14 maize events and 6 cotton events, and S46 contained 7 soybean events and 6 cotton events. Two additional control samples were prepared; S22 with salmon sperm DNA as the no template control (NTC), and S47 with ddH_2_O as the blank control. The single-event DNA solutions at 0.5 ng/μL level were also used to test assay sensitivity.

### Primers and probes

The oligonucleotide primers and fluorescent dye-labeled TaqMan probes were designed according to validated or previously reported methods. The sequences of the primers and probes were those provided in the original reports. The 5′ ends of all TaqMan® fluorescent probes were labeled with the fluorescent reporter 6-carboxy-fluorescein (FAM), and the 3′ ends were labeled with the fluorescent quencher Black Hole Quencher 1 (BHQ1) or Minor Groove Binder Non-fluorescent Quencher (MGBNFQ). All primers and fluorescent probes were synthesized by Shanghai Sangon Biological Engineering Technology and Services Co. Ltd. (Shanghai, China). Table 2 shows details of the primers and probes.

### Real-time PCR

Each TaqMan assay was performed using a CFX96 Real-Time System (Bio-rad, Hercules, CA, USA) in a final volume of 20 µL, containing 20 ng genomic DNA, 1 × TaqMan Universal PCR Master Mix (Applied Biosystems, Foster City, CA, USA), and the primer/probe set. The final concentrations of primers and probes were those described in published reports or standards (Table 2). All real-time PCR reactions were carried out using the following program: a pre-digestion step of 50°C for 2 min; a 95°C initial denaturation and UNG deactivation step for 10 min; 50 cycles of 15 s at 94°C (denaturation), and 1 min at 60°C (annealing and extension). Fluorescence signals were monitored and analyzed at the annealing and extension steps during each PCR cycle using CFX Manager Version 1.6 (Bio-Rad).

### Preamplification

To enrich the amount of template, preamplification was performed for each test sample using the TaqMan PreAmp Master Mix Kit (Life Technologies) according to the manufacturer's protocol, with minor modifications. We included 48 assays in this system. Each primer pair was first diluted to 20 µmol/L with 1 × TE buffer; then, equal volumes (10 µL) of all the primer pairs for the 48 assays were mixed to prepare 480 µL of pooled assay mix. The preamplification PCR reaction mixture contained the following reagents: 50 ng total DNA (containing salmon sperm DNA and GMO DNA) as template, 1× TaqMan® PreAmp Master Mix, 5 µL pooled assay mix, and water to complete the volume to 20 µL. The preamplifications were carried out on a Bio-Rad C1000™ Thermal Cycler (Bio-Rad) using the following protocol from the manufacturer's instructions: initial denaturation at 95°C for 10 min; 14 cycles of 15 s at 95°C (denaturation) and 4 min at 60°C (annealing and extension). Each preamplification product was diluted 20-fold before use as the template in subsequent PCR reactions.

### Real-time PCR on dynamic chips

The Fluidigm 48.48 real-time PCR run was performed according to the manufacturer's instructions (Fluidigm, San Diego, CA, USA). Before performing real-time PCR, the sample mixture and assay mixture were prepared individually. The mixture for each sample (final volume 6 µL) contained 3 µL 2 × TaqMan® Universal PCR Master Mix (Applied Biosystems, PN 4304437), 0.3 µL 20×GE Sample Loading Reagent (Fluidigm, PN 85000746) and 2.7 µL diluted preamplification product as the template. The assay mixture contained 3 µL 2 × Assay Loading Reagent and 3 µL 20 × primer/probe mixture in a final volume of 6 µL. The final concentrations of primers and probes are shown in Table 2. The samples and assay reagents were loaded into separate reaction chambers on the chip on an IFC controller MX (Fluidigm) after adding 5 µL mixture per assay inlet and per sample inlet. The array chip was run on a BioMark HD System (Fluidigm) using a protocol provided in the manufacturer's instructions. The protocol was as follows: a pre-digestion step of 50°C for 2 min; 95°C initial denaturation and UNG deactivation for 10 min; 50 cycles of 15 s at 95°C (denaturation) and 1 min at 60°C (annealing and extension). Fluorescence signals were monitored and analyzed at the annealing and extension steps during every PCR cycle using Q-PCR Analysis Software 3.0.2. (Fluidigm).

## Figures and Tables

**Table 1:** Test of preamplification efficiency by TaqMan assays

Event	TaqMan assays	Ct ValueAfter pre-amplification	Ct ValueBefore preamplification	ΔCt	Mean
BT176	*Bar*	20.01	30.32	10.31	10.10
MON863	*NPTII*	22.87	32.41	9.54	
NK603	NK603	24.15	34.45	10.3	
GTS40-3-2	P-CaMV35S	20.84	31.54	10.7	
LL25	T-NOS	23.26	33.59	10.33	
T45	*PAT*	23.78	34.16	10.38	
OXY-235	OXY-235	25.74	35.31	9.57	
TT51-1	TT51-1	24.16	34.32	10.16	
TT51-1	*Cry1Ab*	22.21	31.86	9.65	

**Table 2:** Primers and probes used in this study

	Assay	Orientation	Sequences (5′-3′)	Final concentration	Amplicon size (bp)	Reference
1	Cotton	F	CACATGACTTAGCCCATCTTTGC	200	76	http://gmo-crl.jrc.ec.europa.eu/gmomethods/docs/QT-EVE-GH-002.pdf
		R	CCCACCCTTTTTTGGTTTAGC	200		
		P	FAM-TGCAGGTTTTGGTGCCACTGTGAATG-BHQ1	200		
2	Maize	F	CGTCGTTTCCCATCTCTTCCTCC	300	135	http://gmo-crl.jrc.ec.europa.eu/gmomethods/docs/QT-EVE-ZM-006.pdf
		R	CCACTCCGAGACCCTCAGTC	300		
		P	FAM-AATCAGGGCTCATTTTCTCGCTCCTCA-BHQ1	200		
3	Rapeseed	F	GGCCAGGGTTTCCGTGAT	200	101	http://gmo-crl.jrc.ec.europa.eu/gmomethods/docs/QT-EVE-BN-001.pdf
		R	CCGTCGTTGTAGAACCATTGG	200		
		P	FAM-AGTCCTTATGTGCTCCACTTTCTGGTGCA-BHQ1	200		
4	Rice	F	TGGTGAGCGTTTTGCAGTCT	200	68	http://gmo-crl.jrc.ec.europa.eu/gmomethods/docs/QT-EVE-OS-002.pdf
		R	CTGATCCACTAGCAGGAGGTCC	200		
		P	FAM-TGTTGTGCTGCCAATGTGGCCTG-BHQ1	200		
5	Soybean	F	CCAGCTTCGCCGCTTCCTTC	150	74	http://gmo-crl.jrc.ec.europa.eu/gmomethods/docs/QT-EVE-GM-005.pdf
		R	GAAGGCAAGCCCATCTGCAAGCC	150		
		P	FAM-CTTCACCTTCTATGCCCCTGACAC-BHQ1	50		
6	*Bar*	F	ACAAGCACGGTCAACTTCC	140	60	Grohmann et al, 2009^39^
		R	GAGGTCGTCCGTCCACTC	140		
		P	FAM-TACCGAGCCGCAGGAACC-BHQ1	100		
7	*Cry1Ab*	F	GGGAAATGCGTATTCAATTCAAC	300	129	http://gmo-crl.jrc.ec.europa.eu/gmomethods/docs/QT-ELE-00-003.pdf
		R	TTCTGGACTGCGAACAATGG	300		
		P	FAM-ACATGAACAGCGCCTTGACCACAGC-BHQ1	160		
8	P-CaMV35S	F	CATCATTGCGATAAAGGAAAGGC	400	125	In laboratory
		R	TGCTTTGAAGACGTGGTTGGA	400		
		P	FAM-TCGTGGGTGGGGGTC-MGBNFQ	200		
9	P-FMV35S	F	AAGACATCCACCGAAGACTTA	200		National standard SN/T 1204-2003^40^
		R	AGGACAGCTCTTTTCCACGTT	200		
		P	FAM-TGGTCCCCACAAGCCAGCTGCTCGA-BHQ1	100		
10	T-NOS	F	ATCGTTCAAACATTTGGCA	200		National standard SN/T 1204-2003^40^
		R	ATTGCGGGACTCTAATCATA	200		
		P	FAM-CATCGCAAGACCGGCAACAGG-BHQ1	100		
11	*NPTII*	F	CTATGACTGGGCACAACAGACA	800	101	Announcement by the Ministry of Agriculture No.1782-2-2012^41^
		R	CGGACAGGTCGGTCTTGACA	800		
		P	FAM-CTGCTCTGATGCCGCCGTGTTCCG-BHQ1	400		
12	*PAT*	F	CGCGGTTTGTGATATCGTTAAC	400		Zeitler et al, 2002^42^
		R	TCTTGCAACCTCTCTAGATCATCAA	400		
		P	FAM-AGGACAGAGCCACAAACACCACAAGAGTG-BHQ1	200		
13	MON531	F	TCCCATTCGAGTTTCTCACGT	150	72	http://gmo-crl.jrc.ec.europa.eu/gmomethods/docs/QT-EVE-GH-004.pdf
		R	AACCAATGCCACCCCACTGA	150		
		P	FAM-TTGTCCCTCCACTTCTTCTC-BHQ1	50		
14	MON88913	F	GGCTTTGGCTACCTTAAGAGAGTC	500	94	http://gmo-crl.jrc.ec.europa.eu/gmomethods/docs/QT-EVE-GH-007.pdf
		R	CAAATTACCCATTAAGTAGCCAAATTAC	500		
		P	FAM-AACTATCAGTGTTTGACTACAT-MGBNFQ	100		
15	MON1445	F	GGAGTAAGACGATTCAGATCAAACAC	150	87	http://gmo-crl.jrc.ec.europa.eu/gmomethods/docs/QT-EVE-GH-003.pdf
		R	ATCGACCTGCAGCCCAAGCT	150		
		P	FAM-ATCAGATTGTCGTTTCCCGCCTTCAGTTT-BHQ1	50		
16	MON15985	F	GTTACTAGATCGGGGATATCC	150	82	http://gmo-crl.jrc.ec.europa.eu/gmomethods/docs/QT-EVE-GH-005.pdf
		R	AAGGTTGCTAAATGGATGGGA	150		
		P	FAM-CCGCTCTAGAACTAGTGGATCTGCACTGAA-BHQ1	50		
17	LLCotton25	F	CAGATTTTTGTGGGATTGGAATTC	400	79	http://gmo-crl.jrc.ec.europa.eu/gmomethods/docs/QT-EVE-GH-002.pdf
		R	CAAGGAACTATTCAACTGAG	400		
		P	FAM-CTTAACAGTACTCGGCCGTCGACCGC-BHQ1	200		
18	GHB614	F	CAAATACACTTGGAACGACTTCGT	400	120	http://gmo-crl.jrc.ec.europa.eu/gmomethods/docs/QT-EVE-GH-006.pdf
		R	GCAGGCATGCAAGCTTTTAAA	400		
		P	FAM-CTCCATGGCGATCGCTACGTTCTAGAATT-BHQ1	200		
19	3272	F	TCATCAGACCAGATTCTCTTTTATGG	50	95	http://gmo-crl.jrc.ec.europa.eu/gmomethods/docs/QT-EVE-ZM-019.pdf
		R	CGTTTCCCGCCTTCAGTTTA	900		
		P	FAM-ACTGCTGACGCGGCCAAACACTG-BHQ1	200		
20	59122	F	GGGATAAGCAAGTAAAAGCGCTC	250	86	http://gmo-crl.jrc.ec.europa.eu/gmomethods/docs/QT-EVE-ZM-012.pdf
		R	CCTTAATTCTCCGCTCATGATCAG	250		
		P	FAM-TTTAAACTGAAGGCGGGAAACGACAA-BHQ1	200		
21	Bt176	F	GGCCGTGAACGAGCTGTT	300	82	http://gmo-crl.jrc.ec.europa.eu/gmomethods/docs/QT-EVE-ZM-023.pdf
		R	GGGAAGAAGCCTACATGTTTTCTAA	600		
		P	FAM-AGCAACCAGATCGGCCGACACC-BHQ1	200		
22	Bt11	F	GCGGAACCCCTATTTGTTTA	750	70	http://gmo-crl.jrc.ec.europa.eu/gmomethods/docs/QT-EVE-ZM-006.pdf
		R	TCCAAGAATCCCTCCATGAG	750		
		P	FAM-AAATACATTCAAATATGTATCCGCTCA-BHQ1	250		
23	GA21	F	CTTATCGTTATGCTATTTGCAACTTTAGA	150	112	http://gmo-crl.jrc.ec.europa.eu/gmomethods/docs/QT-EVE-ZM-007.pdf
		R	TGGCTCGCGATCCTCCT	150		
		P	FAM-CATATACTAACTCATATCTCTTTCTCAACAGCAGGTGGGT-BHQ1	50		
24	MIR 162	F	GCGCGGTGTCATCTATGTTACTAG	300	92	http://gmo-crl.jrc.ec.europa.eu/gmomethods/docs/QT-EVE-ZM-022.pdf
		R	TGCCTTATCTGTTGCCTTCAGA	300		
		P	FAM-TCTAGACAATTCAGTACATTAAAAACGTCCGCCA-BHQ1	150		
25	MIR604	F	GCGCACGCAATTCAACAG	600	76	http://gmo-crl.jrc.ec.europa.eu/gmomethods/docs/QT-EVE-ZM-013.pdf
		R	GGTCATAACGTGACTCCCTTAATTCT	300		
		P	FAM-AGGCGGGAAACGACAATCTGATCATG-BHQ1	200		
26	MON810	F	TCGAAGGACGAAGGACTCTAACGT	300	92	http://gmo-crl.jrc.ec.europa.eu/gmomethods/docs/QT-EVE-ZM-020.pdf
		R	GCCACCTTCCTTTTCCACTATCTT	300		
		P	FAM-AACATCCTTTGCCATTGCCCAGC-BHQ1	180		
27	MON863	F	GTAGGATCGGAAAGCTTGGTAC	150	84	http://gmo-crl.jrc.ec.europa.eu/gmomethods/docs/QT-EVE-ZM-009.pdf
		R	TGTTACGGCCTAAATGCTGAACT	150		
		P	FAM-TGAACACCCATCCGAACAAGTAGGGTCA-BHQ1	50		
28	MON88017	F	GAGCAGGACCTGCAGAAGCT	150	95	http://gmo-crl.jrc.ec.europa.eu/gmomethods/docs/QT-EVE-ZM-016.pdf
		R	TCCGGAGTTGACCATCCA	150		
		P	FAM-TCCCGCCTTCAGTTTAAACAGAGTCGGGT-BHQ1	50		
29	MON89034	F	TTCTCCATATTGACCATCATACTCATT	450	77	http://gmo-crl.jrc.ec.europa.eu/gmomethods/docs/QT-EVE-ZM-018.pdf
		R	CGGTATCTATAATACCGTGGTTTTTAA	450		
		P	FAM-ATCCCCGGAAATTATGTT-MGBNFQ	100		
30	NK603	F	ATGAATGACCTCGAGTAAGCTTGTTAA	150	108	http://gmo-crl.jrc.ec.europa.eu/gmomethods/docs/QT-EVE-ZM-008.pdf
		R	AAGAGATAACAGGATCCACTCAAACACT	150		
		P	FAM-TGGTACCACGCGACACACTTCCACTC-BHQ1	50		
31	T25	F	ACAAGCGTGTCGTGCTCCAC	400	102	http://gmo-crl.jrc.ec.europa.eu/gmomethods/docs/QT-EVE-ZM-011.pdf
		R	GACATGATACTCCTTCCACCG	400		
		P	FAM-TCATTGAGTCGTTCCGCCATTGTCG-BHQ1	200		
32	TC1507	F	TAGTCTTCGGCCAGAATGG	300	58	http://gmo-crl.jrc.ec.europa.eu/gmomethods/docs/QT-EVE-ZM-010.pdf
		R	CTTTGCCAAGATCAAGCG	300		
		P	FAM-TAACTCAAGGCCCTCACTCCG-BHQ1	150		
33	MS1	F	ACGCTGCGGACATCTACATT	400	187	http://gmo-crl.jrc.ec.europa.eu/gmomethods/docs/QT-EVE-BN-005.pdf
		R	CTAGATCGGAAGCTGAAGATGG	400		
		P	FAM-CTCATTGCTGATCCACCTAGCCGACTT-BHQ1	200		
34	Topas 19/2	F	GTTGCGGTTCTGTCAGTTCC	400	95	http://gmo-crl.jrc.ec.europa.eu/gmomethods/docs/QT-EVE-BN-008.pdf
		R	AGTTCCAAACGTAAAACGGCTT	400		
		P	FAM-TCCCGGTCATATATCAGCGCCGGTC-BHQ1	200		
35	OXY-235	F	AGAGAATCGTGAAATTATCTCTACCG	300	105	Wu G. et al, **2008**^43^
		R	ATTGACCATCATACTCATTGCTGA	300		
		P	FAM-CCATGTAGATTTCCCGGACATGAAGCC-BHQ1	150		
36	MS8	F	GTTAGAAAAAGTAAACAATTAATATAGCCGG	400	130	http://gmo-crl.jrc.ec.europa.eu/gmomethods/docs/QT-EVE-BN-002.pdf
		R	GGAGGGTGTTTTTGGTTATC	400		
		P	FAM-AATATAATCGACGGATCCCCGGGAATTC-BHQ1	200		
37	RF3	F	AGCATTTAGCATGTACCATCAGACA	400	139	http://gmo-crl.jrc.ec.europa.eu/gmomethods/docs/QT-EVE-BN-003.pdf
		R	CATAAAGGAAGATGGAGACTTGAG	400		
		P	FAM-CGCACGCTTATCGACCATAAGCCCA-BHQ1	200		
38	RT73	F	CCATATTGACCATCATACTCATTGCT	150	108	http://gmo-crl.jrc.ec.europa.eu/gmomethods/docs/QT-EVE-BN-004.pdf
		R	GCTTATACGAAGGCAAGAAAAGGA	150		
		P	FAM-TTCCCGGACATGAAGATCATCCTCCTT-BHQ1	50		
39	T45	F	CAATGGACACATGAATTATGC	400	123	http://gmo-crl.jrc.ec.europa.eu/gmomethods/docs/QT-EVE-BN-001.pdf
		R	GACTCTGTATGAACTGTTCGC	400		
		P	FAM-TAGAGGACCTAACAGAACTCGCCGT-BHQ1	200		
40	TT51-1	F	GCGTCCAGAAGGAAAAGGAATA	800	120	Wu et al, **2013**^44^
		R	AGAGACTGGTGATTTCAGCGGG	800		
		P	FAM-ATCTGCCCCAGCACTCGTCCG-BHQ1	400		
41	GTS40-3-2	F	TTCATTCAAAATAAGATCATACATACAGGTT	150	84	http://gmo-crl.jrc.ec.europa.eu/gmomethods/docs/QT-EVE-GM-005.pdf
		R	GGCATTTGTAGGAGCCACCTT	150		
		P	FAM-CCTTTTCCATTTGGG-MGBNFQ	50		
42	A2704-12	F	GCAAAAAAGCGGTTAGCTCCT	400	64	http://gmo-crl.jrc.ec.europa.eu/gmomethods/docs/QT-EVE-GM-004.pdf
		R	ATTCAGGCTGCGCAACTGTT	400		
		P	FAM-CGGTCCTCCGATCGCCCTTCC-BHQ1	200		
43	MON 89788	F	TCCCGCTCTAGCGCTTCAAT	150	139	http://gmo-crl.jrc.ec.europa.eu/gmomethods/docs/QT-EVE-GM-006.pdf
		R	TCGAGCAGGACCTGCAGAA	150		
		P	FAM-CTGAAGGCGGGAAACGACAATCTG-BHQ1	50		
44	DP-356043	F	GTCGAATAGGCTAGGTTTACGAAAAA	750	99	http://gmo-crl.jrc.ec.europa.eu/gmomethods/docs/QT-EVE-GM-009.pdf
		R	TTTGATATTCTTGGAGTAGACGAGAGTGT	750		
		P	FAM-CTCTAGAGATCCGTCAACATGGTGGAGCAC-BHQ1	200		
45	A5547-127	F	GCTATTTGGTGGCATTTTTCCA	400	75	http://gmo-crl.jrc.ec.europa.eu/gmomethods/docs/QT-EVE-GM-007.pdf
		R	CACTGCGGCCAACTTACTTCT	400		
		P	FAM-CCGCAATGTCATACCGTCATCGTTGT-BHQ1	200		
46	CV-127	F	AACAGAAGTTTCCGTTGAGCTTTAAGAC	400	88	http://gmo-crl.jrc.ec.europa.eu/gmomethods/docs/QT-EVE-GM-011.pdf
		R	CATTCGTAGCTCGGATCGTGTAC	400		
		P	FAM-TTTGGGGAAGCTGTCCCATGCCC-BHQ1	100		
47	DP-305423	F	CGTGTTCTCTTTTTGGCTAGC	800	93	http://gmo-crl.jrc.ec.europa.eu/gmomethods/docs/QT-EVE-GM-008.pdf
		R	GTGACCAATGAATACATAACACAAACTA	500		
		P	FAM-TGACACAAATGATTTTCATACAAAAGTCGAGA-BHQ1	220		
48	MON87701	F	TGGTGATATGAAGATACATGCTTAGCAT'	600	89	http://gmo-crl.jrc.ec.europa.eu/gmomethods/docs/QT-EVE-GM-010.pdf
		R	CGTTTCCCGCCTTCAGTTTAAA	600		
		P	TCAGTGTTTGACACACACACTAAGCGTGCC	250		
